# Physical activity in advanced cancer patients: a systematic review protocol

**DOI:** 10.1186/s13643-016-0220-x

**Published:** 2016-03-11

**Authors:** Sonya S. Lowe, Maria Tan, Joan Faily, Sharon M. Watanabe, Kerry S. Courneya

**Affiliations:** Department of Symptom Control and Palliative Care, Cross Cancer Institute, 11560 University Avenue, Edmonton, AB T6G 1Z2 Canada; Knowledge Resource Service, Knowledge Management Department, Research Innovation and Analytics, Abdul Khaliq Library, Cross Cancer Institute, 11560 University Avenue, Edmonton, AB T6G 1Z2 Canada; Edmonton Zone Palliative Care Program-Palliative Community Consult Team (EZPCP-PCCT), #335, 1090 Youville Drive West, Edmonton, AB T6L 0A3 Canada; Division of Palliative Care Medicine, Department of Oncology, University of Alberta, Cross Cancer Institute, 11560 University Avenue, Edmonton, AB T6G 1Z2 Canada; Faculty of Physical Education and Recreation, University of Alberta, 1-113 University Hall, Edmonton, AB T6G 2H9 Canada

**Keywords:** Exercise, Oncology, Neoplasms, Terminal illness, End of life, Cancer, Palliative care, Physical activity, Quality of life, Systematic review

## Abstract

**Background:**

Progressive, incurable cancer is associated with increased fatigue, increased muscle weakness, and reduced physical functioning, all of which negatively impact quality of life. Physical activity has demonstrated benefits on cancer-related fatigue and physical functioning in early-stage cancer patients; however, its impact on these outcomes in end-stage cancer has not been established. The aim of this systematic review is to determine the potential benefits, harms, and effects of physical activity interventions on quality of life outcomes in advanced cancer patients.

**Methods/design:**

A systematic review of peer-reviewed literature on physical activity in advanced cancer patients will be undertaken. Empirical quantitative studies will be considered for inclusion if they present interventional or observational data on physical activity in advanced cancer patients. Searches will be conducted in the following electronic databases: CINAHL; CIRRIE Database of International Rehabilitation Research; Cochrane Database of Systematic Reviews (CDSR); Database of Abstracts of Reviews of Effects (DARE); Cochrane Central Register of Controlled Trials (CENTRAL); EMBASE; MEDLINE; PEDro: the Physiotherapy Evidence Database; PQDT; PsycInfo; PubMed; REHABDATA; Scopus; SPORTDiscus; and Web of Science, to identify relevant studies of interest. Additional strategies to identify relevant studies will include citation searches and evaluation of reference lists of included articles. Titles, abstracts, and keywords of identified studies from the search strategies will be screened for inclusion criteria. Two independent reviewers will conduct quality appraisal using the Effective Public Health Practice Project Quality Assessment Tool for Quantitative Studies (EPHPP) and the Cochrane risk of bias tool. A descriptive summary of included studies will describe the study designs, participant and activity characteristics, and objective and patient-reported outcomes.

**Discussion:**

This systematic review will summarize the current evidence base on physical activity interventions in advanced cancer patients. The findings from this systematic review will identify gaps to be explored by future research studies and inform future practice guideline development of physical activity interventions in advanced cancer patients.

**Systematic review registration:**

PROSPERO CRD42015026281

**Electronic supplementary material:**

The online version of this article (doi:10.1186/s13643-016-0220-x) contains supplementary material, which is available to authorized users.

## Background

Quality of life is a subjective, multidimensional construct encompassing several aspects of physical and psychosocial well-being [1]. Optimizing quality of life is the primary goal of palliative care, which the World Health Organization defines as the interdisciplinary and holistic management of progressive, life-threatening disease wherein prognosis is limited [2]. Although it has demonstrated quality of life benefits at earlier stages in the disease trajectory [3], palliative care plays a principal role in the last months of life wherein disease progression is accompanied by a decline in overall quality of life [4].

Cancer is the leading life-threatening disease [5]. It is estimated that, in 2008, 12.7 million new cancer cases were diagnosed and 7.6 million cancer deaths occurred worldwide [6]. In advanced cancer, which can be defined as progressive, incurable, and locally recurrent or metastatic disease, increasing symptom burden and intensity leads to deterioration in global quality of life [7]. In a cross-sectional survey of 3030 patients from European palliative care centers, the two most prevalent symptoms were generalized weakness (50 %) and fatigue (48 %) [8].

Given the negative impact of fatigue and loss of physical functioning on quality of life in cancer patients, recent attention has been given to behavioral interventions to improve these outcomes [9]. Systematic reviews highlight a growing consensus that moderate-to-vigorous-intensity physical activity can improve several aspects of physical and psychological well-being that contribute to quality of life in early-stage cancer patients [10–12]. The American Cancer Society’s most recent guidelines recommend regular exercise to cancer patients both during and after treatment for improved quality of life [13]. These conclusions, however, are drawn from an evidence base that is largely restricted to early-stage cancer patients who are able and willing to participate in moderate-to-vigorous-intensity exercise interventions. Given that disease progression is associated with worsened fatigue, loss of physical functioning, and deterioration in overall quality of life [4], the effect of physical activity on these outcomes in advanced cancer patients is unknown.

Three previous reviews have reported that there was insufficient evidence to determine the safety, feasibility, or efficacy of physical activity interventions in advanced cancer patients [14–16]; an update is required as new literature has since become available. To date, there are no established guidelines on the role of physical activity as a supportive care intervention in this patient population. The most appropriate physical activity program for which type of advanced cancer and level of performance status is not defined. Thus, a systematic review is necessary to update the current evidence base of physical activity interventions in advanced cancer patients. Furthermore, a systematic review is needed to identify gaps to be explored by future research studies and to inform future practice guideline development of physical activity interventions in advanced cancer patients.

### Aims and objectives

The aim of this systematic review is to evaluate the potential benefits, harms, and effects of physical activity interventions on quality of life outcomes in advanced cancer patients. The ultimate goal is to identify the gaps in the existing literature, to develop interventions that can be tested in clinical research, and to inform physical activity recommendations to improve quality of life for advanced cancer patients.

## Methods

### Design

The reporting of this systematic review protocol will adhere to the preferred reporting items for systematic reviews and meta-analyses protocol (PRISMA-P) checklist (see Additional file 1) [17]. The conduct of this systematic review will be guided by AMSTAR [18].

### Types of studies

The studies of interest will be those that examine physical activity as a supportive care intervention in advanced cancer patients. A preliminary scan of the available literature suggests that restriction of the review to randomized controlled trials would not be informative. Empirical research studies using quantitative methods, intervention, or observational design will be included. The types of quantitative study designs to be included will be randomized and non-randomized controlled trials. The following gray literature sources will be searched: ProQuest Dissertations and Theses Global (PQDT), OCLC PapersFirst, European Association of Palliative Care conference abstracts, Multinational Association of Supportive Care in Cancer symposium abstracts, and American College of Sports Medicine meeting abstracts.

### Types of participants

Adults aged 18 years and older will be included as study participants. Lack of consensus on defining the palliative cancer population is a well-recognized limitation in palliative care research; the definitions for “advanced cancer,” “palliative,” “end-stage cancer,” and “terminal illness” are not uniform across studies [19]. For the purposes of this review, advanced cancer is defined as progressive, incurable, locally recurrent, or metastatic malignancy, with an estimated life expectancy limited to 12 months or less. In the situation where a study examines a mixed population of cancer patients at various stages of disease, only those studies wherein data was presented separately for the palliative subgroup of interest will be included. Where feasible, study authors will be contacted when participant eligibility is uncertain.

### Types of interventions/exposures

Any health outcomes research examining physical activity in advanced cancer patients will be included. Physical activity will be defined as any bodily movement produced by the skeletal muscles that results in a substantial increase in energy expenditure over resting levels [20]. For the purposes of this systematic review, basic self-care activities, such as bathing, dressing, and position transfers, are not included in this definition of physical activity. Any study focusing on physical activity preferences, correlates, or behavior change interventions will be excluded.

### Types of comparison

The comparison groups may be no intervention or standard of care.

### Types of outcome measures

Included studies must examine at least one of the following primary outcomes: (1) patient-reported quality of life, (2) patient-reported physical function, or (3) patient-reported fatigue. Secondary outcomes of interest would include the following: (1) objective measures of physical fitness; (2) objective measures of physical function; (3) patient-reported symptoms including pain, depression, and dyspnea; and (4) other adverse outcomes. A preliminary scan of the available literature suggests that the body of evidence may be at risk of outcome reporting bias, which is defined as “the selection for publication of a subset of the original recorded outcome variables on the basis of the results” [21]; therefore, where feasible, study authors will be contacted to obtain results and for confirmation of whether an outcome was measured and analyzed, or if data was incomplete.

### Search strategy

Relevant studies will be searched from the following electronic databases (listed in alphabetical order): Cumulative Index to Nursing and Allied Health Literature (CINAHL); Center for International Rehabilitation Research Information and Exchange (CIRRIE) Database of International Rehabilitation Research; Cochrane Database of Systematic Reviews (CDSR); Database of Abstracts of Reviews of Effects (DARE); Cochrane Central Register of Controlled Trials (CENTRAL); Excerpta Medica Database (EMBASE); Medical Literature Analysis and Retrieval System Online (MEDLINE); PEDro: the Physiotherapy Evidence Database; Psychological Abstracts (PsycINFO); US National Library of Medicine Database (PubMed); National Rehabilitation Information Center Database (REHABDATA); Elsevier Bibliographic Database (Scopus); EBSCO Sports Medicine Database (SPORTDiscus); and Web of Science. Reference lists of all included articles will be hand-searched for additional studies. Additional studies meeting inclusion criteria will be located by assessing the reference lists of relevant reviews. Forward citations of the included articles will be identified. Further studies meeting inclusion criteria will be located in those databases which offer the “related/similar to” function. Searches will be conducted without a language filter, and subsequent analysis will be restricted to English-language articles, with a supplementary appendix list of citations to articles in languages other than English.

The proposed MEDLINE search strategy (Additional file 2) was developed in collaboration with a medical librarian (second author), and refined based on peer feedback from two medical librarians in oncology, and from an expert searcher health librarian accessed through the Peer Review of Electronic Search Strategies (PRESS) Forum. PRESS is “a forum for librarians to obtain peer review of their important searches. It is intended for evidence-based peer review based on research into the aspects of an electronic search that are most important to achieving excellent recall with acceptable precision” [22]. For more information on the PRESS Forum and PRESS evidence-based assessment form, we refer the reader to McGowan et al. [23]. The search strategy includes a combination of relevant subject headings and keywords, which will be modified as required for subsequent databases. There will be no date limits. Searches in all databases will be run within the same week, to ensure data retrieval within the same time period. Search results will be exported to and organized and de-duplicated within Endnote X7 (Thompson Reuters, USA). Duplicate articles will be removed, as the same article may be located in more than one source. If there is more than one article from the same study, different data may be extracted from different articles where relevant. Study authors will be contacted, when possible, for additional papers. A search log will be maintained to record the initial search strategy and subsequent modifications, the databases searched, and details on the identified studies.

### Identification and selection of studies

Initial screening will be performed by the first author, with the third author screening 10 % of articles, in keeping with evidence supporting the reproducibility and reliability of decision-making by more than one reviewer [24]. Initial screening of all the databases will be performed to identify all potentially relevant studies, by screening the resulting titles and abstracts to exclude articles that are clearly irrelevant. Both the first and the third authors will screen the final results; if either or both reviewers feel that the article potentially meets the inclusion criteria or if there is inadequate information to make a decision, full-text copies of the article will be retrieved. Second screening by both the first and the third authors will assess retrieved articles against the defined eligibility criteria for inclusion (see Additional file 3). Reviewers will not be blinded to authors, journal, or results. Studies which have been identified by mutual consent will be included in the review. If there is a disagreement, there will be a discussion with all the authors to reach a consensus. A study selection log will be maintained to record the references for excluded studies and the rationale for excluding them during the screening process. As per the PRISMA guidelines [25], a flow diagram will be developed to report the process of study selection. Full-text studies meeting the inclusion criteria will be imported into Endnote X7 (Thompson Reuters, USA).

### Data extraction and management

For each included study, data will be extracted using a standardized form (see Additional file 4) that has been pilot-tested in a previous systematic review in this area [14]. The extracted data will include the following: (1) study details: published/unpublished, title, authors, source, country, and year of publication; (2) study characteristics: eligibility inclusion/exclusion criteria, setting; (3) population characteristics: number of participants, age, gender, cancer diagnosis, and type of cancer treatment; (4) methods: design/allocation, blinding, sampling, loss to follow-up, recruitment rates, retention rates, and adherence rates; (5) intervention characteristics: type of physical activity, frequency, intensity, duration, program length, supervision; and (6) exposure measure: self-reported outcomes, objective outcomes, total physical activity, recreational physical activity, aerobic activity, self-report scales, and adverse outcomes. Study authors will be contacted, where possible, for missing or incomplete data. The first author will perform data extraction, the results of which will be checked by the third author. The extracted data will be collected in a Microsoft Excel spreadsheet summary to enable comparison between studies. Studies similar in design (observational versus interventional) will be grouped together in the summary to facilitate quality assessment and data synthesis where appropriate.

### Quality assessment

For observational studies, the first and third authors will independently assess the methodological quality according to the Effective Public Health Practice Project Quality Assessment Tool for Quantitative Studies (EPHPP), rating each of the following study components as strong, moderate, or weak: selection bias, study design, confounders, blinding, data collection methods, and withdrawals/dropouts [26]. For randomized controlled trials, the first and third authors will independently evaluate according to low or high risk of bias using the Cochrane risk of bias tool [27] across the following seven domains: sequence generation, allocation concealment, blinding of participants, outcome assessors, incomplete outcome, selective outcome reporting, and other sources. Two different reviewers will be solicited for quality appraisal in the event that either reviewer is an author of an included study. Differences in quality assessment will be resolved by discussion between all authors. The Grading of Recommendations, Assessment, Development and Evaluation (GRADE) framework will be used to determine the strength of evidence through evaluation of risk of bias, imprecision (random error), inconsistency, indirectness, and publication bias [28].

### Data synthesis

Statistical meta-analysis will be done if studies are clinically homogenous and the data permit. In the event that meta-analysis is not possible, narrative synthesis of data will be conducted, following Popay et al.’s framework for narrative synthesis [29]. Data from studies will be grouped according to study characteristics and then collated in a tabulated summary. A comprehensive descriptive account of study quality, strengths, and limitations will be reported. Study recommendation for potential avenues for future research will likewise be reported.

## Discussion

### Strengths and limitations of the review

This review is transparent in its adherence to validated methods and employs a systematic and replicable approach toward searching, screening, appraising, and extracting data from the current evidence base. There are clear inclusion and exclusion criteria, and the search strategy is comprehensive. Including information professionals with advanced search skills in the development of the search strategy will improve accuracy of the search methodology. The involvement of two reviewers in screening, data extraction, and quality appraisal will enhance the reliability of the review’s conclusions. With respect to limitations, reporting bias may be present given judgments made by the reviewers. There may be relevant studies which have non-English titles and abstracts and therefore will be excluded at initial screening.

### Implications for research and dissemination

This systematic review will provide a comprehensive and rigorous evidence base from which future research directions for physical activity can be proposed. It can lead to the development of physical activity interventions and inform clinical recommendations regarding physical activity to improve the quality of life of advanced cancer patients. The findings from this systematic review will be disseminated by scientific peer-reviewed publication and conference presentations.

## Additional files

Additional file 1: PRISMA-P (Preferred Reporting Items for Systematic Review and Meta-Analysis Protocols) 2015 checklist. Recommended items to address in a systematic review protocol. (DOC 82 kb) Additional file 2: MEDLINE search strategy. A search strategy in one database is included. (DOCX 36 kb) Additional file 3: Relevance assessment for inclusion form. Criteria by which studies are evaluated for inclusion and exclusion. (DOCX 17 kb) Additional file 4: Data extraction form. Record of pertinent study characteristics, interventions and outcomes for each included study. (DOCX 20 kb)

## Abbreviations

CDSR: Cochrane Database of Systematic Reviews
CENTRAL: Cochrane Central Register of Controlled Trials
CINAHL: Cumulative Index to Nursing and Allied Health Literature
CIRRIE: Center for International Rehabilitation Research Information and Exchange
DARE: Database of Abstracts of Reviews of Effects
EMBASE: Excerpta Medica Database
EPHPP: Effective Public Health Practice Project Quality Assessment Tool for Quantitative Studies
GRADE: Grading of Recommendations, Assessment, Development and Evaluation
MEDLINE: Medical Literature Analysis and Retrieval System Online
PEDro: Physiotherapy Evidence Database
PQDT: ProQuest Dissertations and Theses Global Database
PRESS: Peer Review of Electronic Search Strategies
PRISMA: preferred reporting items for systematic reviews and meta-analyses
PsycINFO: Psychological Abstracts
PubMed: US National Library of Medicine Database
REHABDATA: National Rehabilitation Information Center Database
Scopus: Elsevier Bibliographic Database
SPORTDiscus: EBSCO Sports Medicine Database
Web of Science: Thomson Reuters Scientific Citation Indexing Service
