# Supplementary figures and images for: Physical activity in advanced cancer patients: a systematic review protocol
Source: Syst Rev. 2016 Mar 11;5:43. doi: 10.1186/s13643-016-0220-x (PMC4788843; doi:10.1186/s13643-016-0220-x)

**APPENDIX B: PROPOSED MEDLINE SEARCH STRATEGY**


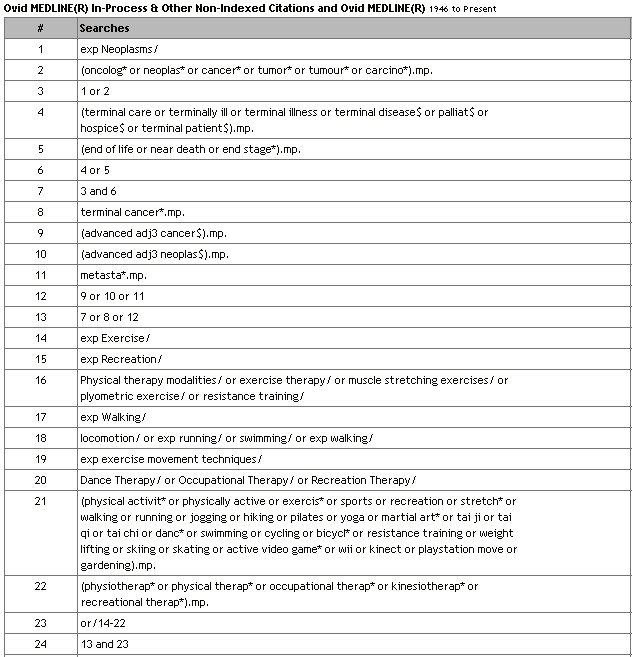

Supplement: Additional file 2: — MEDLINE search strategy. A search strategy in one database is included. (DOCX 36 kb) [file 13643_2016_220_MOESM2_ESM.docx]
