# Supplementary material for: Physical activity in advanced cancer patients: a systematic review protocol
Source: Syst Rev. 2016 Mar 11;5:43. doi: 10.1186/s13643-016-0220-x (PMC4788843; doi:10.1186/s13643-016-0220-x)
Supplement: Additional file 3: — Relevance assessment for inclusion form. Criteria by which studies are evaluated for inclusion and exclusion. (DOCX 17 kb) [file 13643_2016_220_MOESM3_ESM.docx]

**APPENDIX C: RELEVANCE ASSESSMENT FOR INCLUSION**

Physical activity and advanced cancer

**Reviewer:** _____________ **Reference:** ______________ **Date:** _______________

Instructions: Complete the form on each study. If the final decision is “no”, exclude that study.

- 1. **CRITERIA**

|  |  |  |  |  | **Yes** | **No** | **Unsure** |
| --- | --- | --- | --- | --- | --- | --- | --- |
| 1. Study Design | | |  |  |  |  |  |
|  | a. Was the study ***experimental***? | | |  | [ ] | [ ] | [ ] |
|  | (ie. A comparative study in which the use of different interventions among participants is *allocated* by the researcher) | | | |  |  |  |
|  |  | i. Was there random assignment to treatment groups? | | | [ ] | [ ] | [ ] |
|  | b. Was the study ***observational***? | | | | [ ] | [ ] | [ ] |
|  | (ie. A comparative study in which the use of different interventions among participants is not allocated, but merely observed by the researcher) | | | |  |  |  |
|  |  | i. Was there a control group? | | | [ ] | [ ] | [ ] |
|  |  | ii. Was it more than one case? | | | [ ] | [ ] | [ ] |
|  |  |  |  |  |  |  |  |
| 2. Study Population | | |  |  |  |  |  |
|  | a. Did the study include patients aged 18 and over? | | | | [ ] | [ ] | [ ] |
|  | b. Did the patients have the following: | | | |  |  |  |
|  |  | i. Progressive, incurable cancer? | | | [ ] | [ ] | [ ] |
|  |  | ii. Estimated life expectancy less than 12 months? | | | [ ] | [ ] | [ ] |
|  |  |  |  |  |  |  |  |
| 3. Study Intervention | | |  |  |  |  |  |
|  | a. Did the activity involve body movements resulting in increased energy expenditure over rest? | | | | [ ] | [ ] | [ ] |
|  |  |  |  |  |  |  |  |
| 4. Outcome Measures | | |  |  |  |  |  |
|  | a. Did the study consider one or more of the following outcomes: | | | | |  |  |
|  |  | i. Quality of life | |  | [ ] | [ ] | [ ] |
|  |  | ii. Patient-reported physical function | | | [ ] | [ ] | [ ] |
|  |  | iii. Patient-reported fatigue | |  | [ ] | [ ] | [ ] |
|  |  | iv. Objective measures of physical fitness or physical function | | | [ ] | [ ] | [ ] |
|  |  | v. Patient-reported pain, depression or dyspnea | | | [ ] | [ ] | [ ] |
|  |  | vi. Frequency, intensity or duration of physical activity | | | [ ] | [ ] | [ ] |
|  |  | vii. Adverse effects | |  | [ ] | [ ] | [ ] |

1. **DECISION OF REVIEWER**

|  |  |  |  |  | **Yes** | **No** | **Unsure** |
| --- | --- | --- | --- | --- | --- | --- | --- |
| 1. Is this study potentially relevant for this review? | | | | | [ ] | [ ] | [ ] |

1. **CONSENSUS OF ALL REVIEWERS**

First independent review [ ] Include

[ ] Exclude

[ ] Disagree

Re-review by two reviewers [ ] Include

[ ] Exclude

[ ] Disagree

Final consensus by third part adjudication [ ] Include

[ ] Exclude

[ ] Disagree
