# Supplementary material for: Physical activity in advanced cancer patients: a systematic review protocol
Source: Syst Rev. 2016 Mar 11;5:43. doi: 10.1186/s13643-016-0220-x (PMC4788843; doi:10.1186/s13643-016-0220-x)
Supplement: Additional file 4: — Data extraction form. Record of pertinent study characteristics, interventions and outcomes for each included study. (DOCX 20 kb) [file 13643_2016_220_MOESM4_ESM.docx]

**APPENDIX D: DATA EXTRACTION FORM**

**A. GENERAL INFO**

A1. REVIEWER __________________ DATE OF DATA EXTRACTION __________________________

A2. PUBLISHED [ ] UNPUBLISHED [ ]

A3. AUTHOR ________________ TITLE ________________ SOURCE ______________________

YEAR ___________________ VOLUME _____________ PAGES _______________________

INSTITUTION _____________ COUNTRY ___________

**B. STUDY CHARACTERISTICS**

B1. ELIGIBILITY

| STUDY INCLUSION CRITERIA | STUDY EXCLUSION CRITERIA |
| --- | --- |
|  |  |
|  |  |
|  |  |

B2: SETTING

Hospice/Palliative Care Unit [ ]

Cancer Centre [ ]

Hospital [ ]

Community Centre [ ]

Extended Care Facility [ ]

Patient’s Home [ ]

Other ________________ [ ]

Unspecified [ ]

B3. Multi-site (Two or more of B2) [ ] Single site (One of B2) [ ]

B4. POPULATIONS (***NR***: not reported, ***PR***: partially reported)

| Group  (Control: C)  (Intervention: I) | N | Performance Status  (Type) | Performance Status  (Mean and range) | Marital Status  (Type and N%) | Education Level (Type and N/%) | Comorbidity (Type and N/%) | Medication (Type and N/%) | Other |
| --- | --- | --- | --- | --- | --- | --- | --- | --- |
|  |  |  |  |  |  |  |  |  |
|  |  |  |  |  |  |  |  |  |
|  |  |  |  |  |  |  |  |  |
|  |  |  |  |  |  |  |  |  |
|  |  |  |  |  |  |  |  |  |
|  |  |  |  |  |  |  |  |  |
|  |  |  |  |  |  |  |  |  |
|  |  |  |  |  |  |  |  |  |
|  |  |  |  |  |  |  |  |  |
|  |  |  |  |  |  |  |  |  |
|  |  |  |  |  |  |  |  |  |
|  |  |  |  |  |  |  |  |  |
| Total |  |  |  |  |  |  |  |  |

| \| Group  (Control: C)  (Intervention: I) \| N \| Age (mean & range) \| Gender (M:F) \| Diagnosis (Type) \| Diagnosis  (N) \| Cancer Treatment  (Type) (Current or Previous) \| Cancer Treatment  (N) \|  \| \| --- \| --- \| --- \| --- \| --- \| --- \| --- \| --- \| --- \| \|  \|  \|  \|  \|  \|  \|  \|  \|  \| \|  \|  \|  \|  \|  \|  \|  \|  \|  \| \|  \|  \|  \|  \|  \|  \|  \|  \|  \| \|  \|  \|  \|  \|  \|  \|  \|  \|  \| \|  \|  \|  \|  \|  \|  \|  \|  \|  \| \|  \|  \|  \|  \|  \|  \|  \|  \|  \| \|  \|  \|  \|  \|  \|  \|  \|  \|  \| \|  \|  \|  \|  \|  \|  \|  \|  \|  \| \|  \|  \|  \|  \|  \|  \|  \|  \|  \| \| **Total** \|  \|  \|  \|  \|  \|  \|  \|  \| |
| --- | --- | --- | --- | --- | --- | --- | --- | --- | --- | --- | --- | --- | --- | --- | --- | --- | --- | --- | --- | --- | --- | --- | --- | --- | --- | --- | --- | --- | --- | --- | --- | --- | --- | --- | --- | --- | --- | --- | --- | --- | --- | --- | --- | --- | --- | --- | --- | --- | --- | --- | --- | --- | --- | --- | --- | --- | --- | --- | --- | --- | --- | --- | --- | --- | --- | --- | --- | --- | --- | --- | --- | --- | --- | --- | --- | --- | --- | --- | --- | --- | --- | --- | --- | --- | --- | --- | --- | --- | --- | --- | --- | --- | --- | --- | --- | --- | --- | --- | --- |

B4.2. PATIENT MAKE-UP

Consecutive patients [ ]

Random sample [ ]

Convenience sample (by day of week, time, etc.) [ ]

Other ____________ [ ]

Unknown [ ]

B4.3. Eligibility rate (#eligible / total #screened): _____________

| Ineligible (N) | Reasons for ineligibility |
| --- | --- |
|  |  |
|  |  |
|  |  |
|  |  |

B4.4. Recruitment rate (#agreed to participate / #eligible): ______________

| Refused to participate (N) | Reasons for refusal |
| --- | --- |
|  |  |
|  |  |
|  |  |
|  |  |

B4.5. Enrollment rate (#started study / #agreed to participate): _____________

| Not enrolled (N) | Reasons for not enrolling |
| --- | --- |
|  |  |
|  |  |
|  |  |
|  |  |

B4.6. Description of withdrawals (participants who do not fully comply with the intervention, receive an alternative intervention, choose to drop out or are lost to followup):

| Group  (Control: C)  (Intervention: I) | Number of Withdrawals (N) | Reasons for Withdrawals |
| --- | --- | --- |
|  |  |  |
|  |  |  |
|  |  |  |
|  |  |  |
|  |  |  |
|  |  |  |
|  |  |  |

B4.7. Retention rate (#post-intervention assessments / #enrolled): ___________

Adherence rate: (#sessions completed / #sessions prescribed): ___________

B4.8. Blinding

Providers [ ]

Patients [ ]

Outcome Assessors [ ]

Neither/not stated [ ]

B4.9. Other issues:

**C. INTERVENTIONS**

Well described [ ]

|  | Type and Description  (total, recreational, aerobic) | Where performed | Frequency (#sessions per week) | Intensity  Description | Duration per session | Total length of program | Supervision |
| --- | --- | --- | --- | --- | --- | --- | --- |
| Physical Activity |  |  |  |  |  |  |  |
| Control/Standard of Care |  |  |  |  |  |  |  |

1. **OUTCOMES**

D1. Self-reported outcomes

| Outcomes | Name of Tool / Scale | Validity  (known or referenced) | Reliability  (known or referenced) | Sensitivity  (known or referenced) | Time | | Time | | Final | | Other Comment |
| --- | --- | --- | --- | --- | --- | --- | --- | --- | --- | --- | --- |
|  |  |  |  |  | Tx | Con | Tx |  | Con | Tx |  |
| Quality of Life |  |  |  |  |  | |  | |  | |  |
| Physical Function |  |  |  |  |  | |  | |  | |  |
| Fatigue |  |  |  |  |  | |  | |  | |  |
| Pain |  |  |  |  |  | |  | |  | |  |
| Depression |  |  |  |  |  | |  | |  | |  |
| Dyspnea |  |  |  |  |  | |  | |  | |  |
| Other |  |  |  |  |  | |  | |  | |  |

D2. Objective outcomes

| Outcomes | Description | Validity | Reliability | Sensitivity | Time | | Time | | Final | |
| --- | --- | --- | --- | --- | --- | --- | --- | --- | --- | --- |
|  |  |  |  |  | Tx | Con | Tx | Con | Tx | Con |
| Physical Fitness |  |  |  |  |  | |  | |  | |
| Physical Function |  |  |  |  |  | |  | |  | |
| Other |  |  |  |  |  | |  | |  | |

D3. Adverse Outcomes (NR: not reported)

| Adverse Outcomes | Time | | Time | | Final | |
| --- | --- | --- | --- | --- | --- | --- |
|  | Tx | Con | Tx | Con | Tx | Con |
|  |  | |  | |  | |
|  |  | |  | |  | |
|  |  | |  | |  | |

D4. Relevant References:

__________________________________________________________________________________________________________________________________________________________________________________________________________________________________________________________________

D5. Feasibility / Comments:

__________________________________________________________________________________________________________________________________________________________________________________________________________________________________________________________________
